# Microsporidia Detection and Genotyping Study of Human Pathogenic *E. bieneusi* in Animals from Spain

**DOI:** 10.1371/journal.pone.0092289

**Published:** 2014-03-20

**Authors:** Ana Luz Galván-Díaz, Angela Magnet, Soledad Fenoy, Nuno Henriques-Gil, María Haro, Francisco Ponce Gordo, Guadalupe Miró, Carmen del Águila, Fernando Izquierdo

**Affiliations:** 1 Facultad de Farmacia, Universidad San Pablo CEU, Boadilla del Monte, Madrid, Spain; 2 Escuela de Microbiología, Grupo de Parasitología, Universidad de Antioquia, Medellín, Colombia; 3 Facultad de Medicina, Universidad San Pablo CEU, Boadilla del Monte, Madrid, Spain; 4 Facultad de Farmacia, Universidad Complutense de Madrid, Madrid, Madrid, Spain; 5 Facultad de Veterinaria, Universidad Complutense de Madrid, Madrid, Madrid, Spain; Tulane University School of Public Health and Tropical Medicine, United States of America

## Abstract

Microsporidia are ubiquitous parasites infecting all animal phyla and we present evidence that supports their zoonotic potential. Fecal samples taken from domestic (cats and dogs), farm (pigs, rabbits and ostriches) and wild animals (foxes) from different provinces of Spain were evaluated for microsporidia infection by light microscopy and PCR. After Microsporidia species identification, *E. bieneusi* genotypes were additionally studied by sequence analysis of the ITS region. Eighty-five samples out of 159 exhibited structures that were compatible with microsporidia spores by Webeŕs stain with 37 of them being confirmed by PCR. Microsporidia species identified included *E. bieneusi*, *E. intestinalis* and *A. algerae*. We report the first diagnosis of *E. intestinalis* and *E. bieneusi* in ostriches and *A. algerae* in pigs. We also provide new information on the molecular characterization of *E. bieneusi* isolates both in rabbits and ostriches. All of the *E. bieneusi* genotypes identified belonged to the zoonotic group of genotypes (Group I) including genotypes A (dogs), I (pigs), D (rabbits and foxes) and type IV (ostriches). Our results demonstrate that microsporidia are present in domestic, farm and wild animals in Spain, corroborating their potential role as a source of human infection and environmental contamination.

## Introduction

Microsporidia are a ubiquitous group of obligate intracellular parasites that infect all major animal groups (both vertebrates and invertebrates). Transmission occurs mainly through fecal-oral routes, with sources of infection including other infected humans and animals [1], contaminated water [2], [3], [4], and food [1]. Thus far, at least 14 microsporidian species are considered to be pathogenic for humans, *Enterocytozoon bieneusi* being the most prevalent species in humans worldwide followed by *Encephalitozoon intestinalis* [1], [5], [6], [7]. Gastrointestinal tract is the main localization of these parasites with chronic diarrhea as the most frequent clinical manifestation.

Several microsporidia species have been confirmed in a broad range of animals [8], [9], suggesting a zoonotic potential. Although the epidemiology of microsporidian infections still remains unclear, application of molecular techniques based on PCR and sequence analysis, both for species identification and within species genotyping, has increased the available information of microsporidia genetic diversity and it has also proven to be useful in the identification of route(s) and source(s) of transmission of these parasites. In the case of *E. bieneusi*, sequence analysis of the internal transcribed spacer (ITS) region has revealed a considerable genetic variation within *E. bieneusi* isolates of human and animal origins, with more than 100 genotypes described so far [7], [9], [10], [11]. These studies have identified *E. bieneusi* genotypes host-associated to several animals and also some genotypes with no host specificity, which are considered zoonotic [12], [13], [14]. Henriques-Gil et al. [15] identified 4 groups (I-IV) of genotypes strongly differentiated from each other. Group I (zoonotic group) included most of the published sequences (94%), with genotypes that are associated with nearly all human infections [15]. The occasional sequences included in the divergent groups II, III, and IV have so far been restricted to specific hosts such as raccoons (III) and cats and dogs (IV).

In Spain, microsporidia have been confirmed in humans, animals and environmental samples, indicating that these parasites could be more frequent than previously thought and that they should be considered as a potential public health problem. Both Immunocompromised and immunocompetent populations are affected with this infection, with data on microsporidia presence not only in HIV/AIDS patients [16], [17], [18] but also in HIV-negative patients including travelers [19], the elderly [20], organ transplant recipients [21], Crohn’s disease patients [22] and the immunocompetent population [23]. Similar to data described worldwide, *E. bieneusi* is the species most frequently identified. Animals hosts including dogs, goats and rabbits [24], [25], pigeons [26], [27] and soil and fecal samples (presumably from cats and dogs ) from urban parks [28] have also been described for microsporidia in several regions of Spain, with *E. bieneusi* and *Encephalitozoon hellem* as the species identified. All these data suggest that human pathogenic microsporidia circulate in the environment in Spain and support the idea that the most frequent microsporidia associated with human infection are of zoonotic origin.

In this study, we investigate the presence of microsporidia spores in fecal samples from domestic and wild animals from Spain using microscopic and molecular methods. Additionally, taking into account that there is little data on molecular characterization of microsporidia in animal samples in the country, we also studied the genetic diversity of *E. bieneusi* from animal sources including domestic, farm and wild animals, through the sequence analysis of the ITS region. Our results should contribute to the knowledge of the molecular epidemiology of this microsporidia in Spain.

## Materials and Methods

### Ethics Statement

No ethical approval was required for the described study. Endangered or protected species were not included in this work and no specific permissions were necessary for sample collection. Fecal samples were obtained after authorization of the land and animal owners. No animals were harmed in the acquisition of fecal samples.

### Animal stool samples and staining method

**Figure 1:**
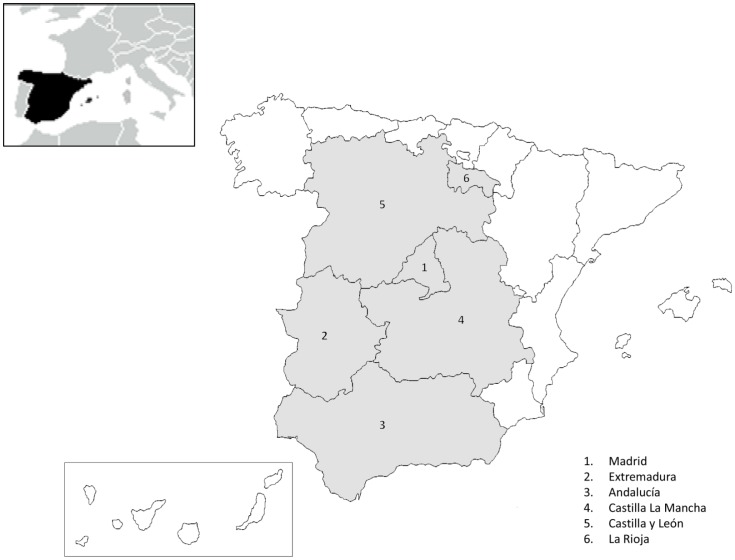
Geographic distribution of the samples included in the study. Animal samples from six different autonomous communities of Spain were evaluated.

A total of 159 fecal samples from domestic (cats and dogs), farm (rabbits, pigs and ostriches) and wild (foxes) animals from different autonomous communities of Spain were evaluated for the presence of microsporidia (Figure 1). All samples were apparently normal with no signs of intestinal disorders. Fecal samples were collected by using a sterile spatula and gloves, and placed into labeled sterile container. The samples were stained with Weber’s chromotrope stain [29]. Microscopic analysis was performed with a magnification of 1000 X and positive criteria included spores with a bright pinkish red stain and either a clear vacuole-like polar end or a belt-like stripe in the middle of the spore.

### DNA extraction and purification

DNA from fecal samples that were positive for microsporidia with the staining method was extracted by bead disruption of spores using the Fast-DNA-Spin soil kit according to the protocol described by Da Silva et al. [30], with a previous 10 minutes incubation step with PVP (polyvinylpyrrolidone). PCR inhibitors were removed using NucleoSpin Extract II kit (MACHEREY-NAGEL GmbH & Co. KG, Germany) according to the manufacturer’s instructions.

### Species identification by PCR

PCR was performed using different diagnostic primer pairs. Generic microsporidia primers pair MicR1 and MicF1 were used to confirm the presence of microsporidia [31]; microsporidial small subunit rRNA (SSU-rRNA) coding regions were amplified using the following species-specific primers: EBIEF1/EBIER1 for *E. bieneusi* [32], SINTF/SINTR for *E. intestinalis* [33], ECUNF/ECUNR for *Encephalitozoon cuniculi* [34], EHELF/EHELR for *E. hellem* [35], NALGf2/NALGR1 for *Anncaliia algerae* [36], and NCORF1/NCORR1 for *Vittaforma corneae* [37]. The PCR amplifications were performed with a GenAmp kit (Perkin-Elmer Cetus, Norwalk, CT) according to the manufacturer’s procedures with a final concentration of 0.2 mM of each dNTP, 0.2 μM of each primer, buffer with MgCl_2_ 1X and 1.25 U of Taq polymerase. PCR was performed in a Gene Amp PCR system 9700 thermocycler (Perkin Elmer) following the amplification protocol described for each species. PCR inhibitors were evaluated by spiking the samples with positive DNA control.

### *E. bieneusi* genotyping

*E. bieneusi* genotyping was performed by sequence analysis of the Internal Transcribed Spacer (ITS) region of rDNA. For this purpose, a fragment of 536 bp containing the 243 bp of the ITS was amplified according to the conditions described by Galván et al. [21].

### DNA sequence analysis

PCR product was purified with QIAquick PCR kit (Qiagen, Chatsworth, CA) and sequenced in both directions through *Macrogen* laboratories (Korea) sequencing service. The resulting sequences were edited and aligned with the Bioedit Sequence Alignment Editor 7.0.5.3 [38].

## Results and Discussion

Eighty-five samples of 159 exhibited structures that were compatible with microsporidia spores by Webeŕs stain with 37 of them being confirmed by PCR. Microsporidia distribution data obtained by both techniques showed that dogs and pigs had the highest number of positive samples for these parasites (Table 1). The differences between the stain and PCR results could be explained by: 1- the presence of PCR inhibitors in the feces; this is a difficult sample matrix which has been shown to contain abundant inhibitors frequently co-purified with fecal DNA and which can, therefore, cause false negative detection results. 2-the presence of spores belonging to other microsporidia that are not detected with the generally group-specific PCR used in our study; this should not be surprising since there have been more than 150 genera described so far, with strong genetic divergence among them.

**Table 1:** Microsporidia species and *E. bieneusi* genotypes in animals from different regions of Spain.

Animal (N)	Autonomous community	Weber’s stain n	PCR phylum n	Microsporidia species (n)	*E. bieneusi* genotype (GenBank accession number)	*E. bieneusi* genotype group^a^
Dogs (73)	Madrid	32	14	*E. bieneusi* (7)	A (AF101197)	I
Cats (9)	Madrid	4	1	*E. intestinalis* (1)	-	-
Pigs (34)	Extremadura Castilla y León	27	15	*E. bieneusi* (7)	I^b^ (AF135836)	I
				*E. intestinalis* (2)	-	-
				*A. algerae* (1)	-	-
Rabbits (19)	Castilla y León^c^ La Rioja^c^ Extremadura^c^ Madrid	4	4	*E. bieneusi* (4)	D^b^ (AF023245)	I
Ostriches (17)	Castilla La Mancha	14	2	*E. bieneusi* (1)	Type IV (AF267141)	I
				*E. intestinalis* (1)	-	-
Foxes (7)	Andalucía	4	1	*E. bieneusi* (1)	D (AF023245)	I

N: Total number of samples; n: number of positive samples.

a According to Henriques-Gil et al. [15].

b Only one sample was successfully genotyped.

c Samples from these provinces were collected from rabbit cages with 5 to 7 animals each one.

*Enterocytozoon bieneusi* was the most commonly detected species (20 out of 37 samples), followed by *E. intestinalis* (4 out of 37 samples) and *A. algerae* (1 out of 37 samples). Undetermined species of microsporidia were detected in 14 out of the 37 samples indicating that additional species not associated with human disease were also identified in the animals examined here. Although most of the animals were infected by a single species, two pigs exhibited a co-infection. The high frequency of *E. bieneusi* is not surprising since a large variety of vertebrate hosts are known to be infected by this species, including humans, other mammals and birds. Consequently, any of them may act as potential reservoirs that facilitate the environmental pollution and continuous transmission of this microsporidia [9]. These findings are in agreement with several studies on microsporidia presence in animals from different regions of Spain, with most of them describing *E. bieneusi* as the main species identified in animals including dogs and goats [25]; pigeons [26] and dogs and rabbits [24]. In the case of *E. intestinalis*, despite being one of the most common microsporidian species found in humans worldwide, it has been less frequently identified in host animals compared to *E. bieneusi* [8]. In our study, even though the number of animals evaluated and positive samples for *E. intestinalis* was lower, the data obtained confirm its presence in domestic animals such as cats and pigs, and contribute to the current knowledge of the spectrum of animal hosts for this parasite, increasing the number of potential sources of infection for humans. It is also important to highlight that, to our knowledge; this is the first diagnosis of *E. intestinalis* and *E. bieneusi* in ostriches. There are only two previous reports of microsporidia presence in these animals, with *E. hellem* as the only microsporidia species identified [39], [40]. *A. algerae* primarily infects moquitoes, but has been reported in humans [41], [42], [43]. We believe this is the first identification of *A. algerae* in another mammal, namely pigs.

*Enterocytozoon bieneusi* is a complex species with multiple genotypes and diverse host range and pathogenicity [7], [9]. The genetic data on *E. bieneusi* diversity rely almost exclusively on the analysis of the ITS region, which provides valuable information about the transmission and pathogenic potential of this parasite. In our study, the sequence analysis of this region showed 4 distinct genotypes (Table 1). All of the genotypes belonged to Group I as previously defined by Henriques-Gil et al. [15]. In the case of dogs, all *E. bieneusi* positive samples (7) had genotype A (GenBank accession no. AF101197), which has long been known as a human specific genotype [9], [11], with only one report of its presence in birds from the Czech Republic [44]. This finding could suggest either that dogs can also be infected from a human source, or, that both, humans and dogs could be infected from the same environmental source. Indeed, other studies have revealed a diverse group of genotypes for *E. bieneusi* isolated from dogs, cats and other pets occasionally analyzed. Zoonotic transmission between dogs and humans is possible for genotypes D, Peru6, WL11, type IV [9,_ENREF_40 45] and, according to our results, also for the genotype A. However, more samples from these animals should be genotyped to confirm the zoonotic transmission of genotype A. By contrast, a dog-specific genotype, PtEb IX [9], [11] corresponding to the group II of sequences that are divergent to the vast majority of *E. bieneusi* ITS genotypes [15], could have an exclusive dog-to-dog transmission.

Regarding *E. bieneusi* diversity in the other animals included in our study, only one sample could be successfully genotyped from each animal species (pigs, rabbits, ostriches and foxes). Genotype I identified in swine has commonly been reported in livestock animals (cattle) [7], [9], [46] and more recently in humans (symptomatic children) [46], but so far there are no reports of its presence in pigs [9]. More studies should be performed in order to confirm that this genotype could be considered relevant in pigs. Henriques-Gil et al. [15] showed that genotypes obtained from swine or cattle are significantly segregated, though some of them may appear in these hosts, plus humans and other domestic animals.

Genotype D (GenBank accession no. AF023245) was detected in both fox and rabbits. This is the genotype that has the widest distribution, and has been found in virtually all host species so far analyzed, including humans, domestic and wild animals, such as foxes [7], [9], [10], [14]. This is the first report in rabbits, and should not be surprising as genotype D probably represents one of the most ancient ITS genotypes in *E. bieneusi* [15]. In the case of the ostriches, we identified the type IV genotype in the only sample that was positive for this microsporidia. Like the previous one, this genotype also occupies a central position in the haplotype network described by Henriques-Gil et al. [15], and likewise it has been found in animals and humans, suggesting that these birds can be included in the extensive network of potential reciprocal *E. bieneusi* infections.

## Conclusion

This study shows that human pathogenic microsporidia species are present in domestic, farm and wild animals in Spain, corroborating their potential role as a source of human infection and environmental contamination. However, larger studies are needed to confirm that animal contact is a high risk for human infection. Our findings demonstrate that *E. bieneusi* genotypes of zoonotic importance are circulating in animals that are in close contact with humans, such as dogs and pigs, and therefore could participate in the transmission of this microsporidia in different regions of Spain. Furthermore, the identification of human classical *E. bieneusi* genotype (genotype A in dogs) suggests the absence of a microsporidia transmission barrier between humans and these animals. Additionally, since *E. bieneusi* is a cause of significant human disease in immunocompromised patients (HIV-infected patients and solid-organ transplant recipients), children and the elderly, among others, our results must be considered in order to establish prophylactic measures that could reduce its transmission.

## References

[1] Didier ES, Stovall ME, Green LC, Brindley PJ, Sestak K, et al (2004) Epidemiology of microsporidiosis: sources and modes of transmission. Vet Parasitol 126: 145–166. 15567583 10.1016/j.vetpar.2004.09.006

[2] Izquierdo F, Castro Hermida JA, Fenoy S, Mezo M, Gonzalez-Warleta M, et al (2011) Detection of microsporidia in drinking water, wastewater and recreational rivers. Water Res 45: 4837–4843. 21774958 10.1016/j.watres.2011.06.033

[3] Magnet A, Galvan AL, Fenoy S, Izquierdo F, Rueda C, et al.. (2012) Molecular characterization of Acanthamoeba isolated in water treatment plants and comparison with clinical isolates. Parasitol Res: 383–392. 10.1007/s00436-012-2849-2 22395660

[4] Galvan AL, Magnet A, Izquierdo F, Fenoy S, Rueda C, et al (2013) Molecular characterization of human-pathogenic microsporidia and Cyclospora cayetanensis isolated from various water sources in Spain: a year-long longitudinal study. Appl Environ Microbiol 79: 449–459. 23124243 10.1128/AEM.02737-12 PMC3553776

[5] Anane S, Attouchi H (2010) Microsporidiosis: epidemiology, clinical data and therapy. Gastroenterol Clin Biol 34: 450–464. 20702053 10.1016/j.gcb.2010.07.003

[6] Didier ES, Weiss LM (2006) Microsporidiosis: current status. Curr Opin Infect Dis 19: 485–492. 16940873 10.1097/01.qco.0000244055.46382.23 PMC3109650

[7] Matos O, Lobo ML, Xiao L (2012) Epidemiology of Enterocytozoon bieneusi Infection in Humans. J Parasitol Res 2012: 981424. 23091702 10.1155/2012/981424 PMC3469256

[8] Mathis A, Weber R, Deplazes P (2005) Zoonotic potential of the microsporidia. Clin Microbiol Rev 18: 423–445. 16020683 10.1128/CMR.18.3.423-445.2005 PMC1195965

[9] Santin M, Fayer R (2011) Microsporidiosis: Enterocytozoon bieneusi in domesticated and wild animals. Res Vet Sci 90: 363–371. 20699192 10.1016/j.rvsc.2010.07.014

[10] Li W, Cama V, Feng Y, Gilman RH, Bern C, et al (2012) Population genetic analysis of Enterocytozoon bieneusi in humans. Int J Parasitol 42: 287–293. 22534008 10.1016/j.ijpara.2012.01.003

[11] Santin M, Fayer R (2009) Enterocytozoon bieneusi genotype nomenclature based on the internal transcribed spacer sequence: a consensus. J Eukaryot Microbiol 56: 34–38. 19335772 10.1111/j.1550-7408.2008.00380.x

[12] Sulaiman IM, Fayer R, Lal AA, Trout JM, Schaefer FW 3rd, et al (2003) Molecular characterization of microsporidia indicates that wild mammals Harbor host-adapted Enterocytozoon spp. as well as human-pathogenic Enterocytozoon bieneusi. Appl Environ Microbiol 69: 4495–4501. 12902234 10.1128/AEM.69.8.4495-4501.2003 PMC169096

[13] Sulaiman IM, Fayer R, Yang C, Santin M, Matos O, et al (2004) Molecular characterization of Enterocytozoon bieneusi in cattle indicates that only some isolates have zoonotic potential. Parasitol Res 92: 328–334. 14727187 10.1007/s00436-003-1049-5

[14] Thellier M, Breton J (2008) Enterocytozoon bieneusi in human and animals, focus on laboratory identification and molecular epidemiology. Parasite 15: 349–358. 18814706 10.1051/parasite/2008153349

[15] Henriques-Gil N, Haro M, Izquierdo F, Fenoy S, del Aguila C (2010) Phylogenetic approach to the variability of the microsporidian Enterocytozoon bieneusi and its implications for inter- and intrahost transmission. Appl Environ Microbiol 76: 3333–3342. 20228101 10.1128/AEM.03026-09 PMC2869117

[16] del Aguila C, Lopez-Velez R, Fenoy S, Turrientes C, Cobo J, et al (1997) Identification of Enterocytozoon bieneusi spores in respiratory samples from an AIDS patient with a 2-year history of intestinal microsporidiosis. J Clin Microbiol 35: 1862–1866. 9196210 10.1128/jcm.35.7.1862-1866.1997 PMC229858

[17] del Aguila C, Moura H, Fenoy S, Navajas R, Lopez-Velez R, et al (2001) In vitro culture, ultrastructure, antigenic, and molecular characterization of Encephalitozoon cuniculi isolated from urine and sputum samples from a Spanish patient with AIDS. J Clin Microbiol 39: 1105–1108. 11230434 10.1128/JCM.39.3.1105-1108.2001 PMC87880

[18] del Aguila C, Navajas R, Gurbindo D, Ramos JT, Mellado MJ, et al (1997) Microsporidiosis in HIV-positive children in Madrid (Spain). J Eukaryot Microbiol 44: 84S–85S. 9580074 10.1111/j.1550-7408.1997.tb05798.x

[19] Lopez-Velez R, Turrientes MC, Garron C, Montilla P, Navajas R, et al (1999) Microsporidiosis in travelers with diarrhea from the tropics. J Travel Med 6: 223–227. 10575169 10.1111/j.1708-8305.1999.tb00522.x

[20] Lores B, Lopez-Miragaya I, Arias C, Fenoy S, Torres J, et al (2002) Intestinal microsporidiosis due to Enterocytozoon bieneusi in elderly human immunodeficiency virus—negative patients from Vigo, Spain. Clin Infect Dis 34: 918–921. 11880956 10.1086/339205

[21] Galvan AL, Sanchez AM, Valentin MA, Henriques-Gil N, Izquierdo F, et al (2011) First cases of microsporidiosis in transplant recipients in Spain and review of the literature. J Clin Microbiol 49: 1301–1306. 21325545 10.1128/JCM.01833-10 PMC3122787

[22] Andreu-Ballester JC, Garcia-Ballesteros C, Amigo V, Ballester F, Gil-Borras R, et al (2013) Microsporidia and its relation to Crohn's disease. A retrospective study. PLoS One 8: e62107. 23637975 10.1371/journal.pone.0062107 PMC3630148

[23] Abreu-Acosta N, Lorenzo-Morales J, Leal-Guio Y, Coronado-Alvarez N, Foronda P, et al (2005) Enterocytozoon bieneusi (microsporidia) in clinical samples from immunocompetent individuals in Tenerife, Canary Islands, Spain. Trans R Soc Trop Med Hyg 99: 848–855. 16111728 10.1016/j.trstmh.2005.05.010

[24] del Aguila C, Izquierdo F, Navajas R, Pieniazek NJ, Miro G, et al (1999) Enterocytozoon bieneusi in animals: rabbits and dogs as new hosts. J Eukaryot Microbiol 46: 8S–9S. 10519225

[25] Lores B, del Aguila C, Arias C (2002) Enterocytozoon bieneusi (microsporidia) in faecal samples from domestic animals from Galicia, Spain. Mem Inst Oswaldo Cruz 97: 941–945. 12471418 10.1590/s0074-02762002000700003

[26] Haro M, Henriques-Gil N, Fenoy S, Izquierdo F, Alonso F, et al (2006) Detection and genotyping of Enterocytozoon bieneusi in pigeons. J Eukaryot Microbiol 53 Suppl 1S58–60. 17169068 10.1111/j.1550-7408.2006.00173.x

[27] Haro M, Izquierdo F, Henriques-Gil N, Andres I, Alonso F, et al (2005) First detection and genotyping of human-associated microsporidia in pigeons from urban parks. Appl Environ Microbiol 71: 3153–3157. 15933015 10.1128/AEM.71.6.3153-3157.2005 PMC1151808

[28] Dado D, Izquierdo F, Vera O, Montoya A, Mateo M, et al (2012) Detection of zoonotic intestinal parasites in public parks of Spain. Potential epidemiological role of microsporidia. Zoonoses Public Health 59: 23–28. 21824364 10.1111/j.1863-2378.2011.01411.x

[29] Weber R, Bryan RT, Owen RL, Wilcox CM, Gorelkin L, et al (1992) Improved light-microscopical detection of microsporidia spores in stool and duodenal aspirates. The Enteric Opportunistic Infections Working Group. N Engl J Med 326: 161–166. 1370122 10.1056/NEJM199201163260304

[30] da Silva AJ, Bornay-Llinares FJ, Moura IN, Slemenda SB, Tuttle JL, et al (1999) Fast and reliable extraction of protozoan parasite DNA from fecal specimens. Mol Diagn 4: 57–64. 10229775 10.1016/s1084-8592(99)80050-2

[31] Dowd SE, John D, Eliopolus J, Gerba CP, Naranjo J, et al (2003) Confirmed detection of Cyclospora cayetanesis, Encephalitozoon intestinalis and Cryptosporidium parvum in water used for drinking. J Water Health 1: 117–123. 15384722

[32] da Silva AJ, Schwartz DA, Visvesvara GS, de Moura H, Slemenda SB, et al (1996) Sensitive PCR diagnosis of Infections by Enterocytozoon bieneusi (microsporidia) using primers based on the region coding for small-subunit rRNA. J Clin Microbiol 34: 986–987. 8815125 10.1128/jcm.34.4.986-987.1996 PMC228934

[33] Da Silva AJ, Slemenda SB, Visvesvara GS, Schwartz DA, Wilcox CM, et al (1997) Detection of Septata intestinalis (Microsporidia) Cali et al. 1993 Using Polymerase Chain Reaction Primers Targeting the Small Submit Subunit Ribosomal RNA Coding Region. Mol Diagn 2: 47–52. 10462591 10.1054/MODI00200047

[34] De Groote MA, Visvesvara G, Wilson ML, Pieniazek NJ, Slemenda SB, et al (1995) Polymerase chain reaction and culture confirmation of disseminated Encephalitozoon cuniculi in a patient with AIDS: successful therapy with albendazole. J Infect Dis 171: 1375–1378. 7751721 10.1093/infdis/171.5.1375

[35] Visvesvara GS, Leitch GJ, da Silva AJ, Croppo GP, Moura H, et al (1994) Polyclonal and monoclonal antibody and PCR-amplified small-subunit rRNA identification of a microsporidian, Encephalitozoon hellem, isolated from an AIDS patient with disseminated infection. J Clin Microbiol 32: 2760–2768. 7852569 10.1128/jcm.32.11.2760-2768.1994 PMC264156

[36] Visvesvara GS, Belloso M, Moura H, Da Silva AJ, Moura IN, et al (1999) Isolation of Nosema algerae from the cornea of an immunocompetent patient. J Eukaryot Microbiol 46: 10S. 10519226

[37] Ghosh K, Weiss LM (2009) Molecular diagnostic tests for microsporidia. Interdiscip Perspect Infect Dis 2009: 926521. 19657457 10.1155/2009/926521 PMC2719812

[38] Hall TA (1999) BioEdit: a user-friendly biological sequence alignment editor and analysis program for Windows 95/98/NT. Nucl Acids Symp Ser 41: 95–98.

[39] Gray ML, Puette M, Latimer KS (1998) Microsporidiosis in a young ostrich (Struthio camelus). Avian Dis 42: 832–836. 9876859

[40] Snowden K, Logan K (1999) Molecular identification of encephalitozoon hellem in an ostrich. Avian Dis 43: 779–782. 10611995

[41] Cali A, Neafie R, Weiss LM, Ghosh K, Vergara RB, et al (2010) Human vocal cord infection with the Microsporidium Anncaliia algerae. The Journal of eukaryotic microbiology 57: 562–567. 20958855 10.1111/j.1550-7408.2010.00510.x PMC3109663

[42] Cali A, Weiss LM, Takvorian PM (2005) A review of the development of two types of human skeletal muscle infections from microsporidia associated with pathology in invertebrates and cold-blooded vertebrates. Folia parasitologica 52: 51–61. 16004364 10.14411/fp.2005.007 PMC3109649

[43] Cali A, Weiss LM, Takvorian PM (2004) An analysis of the microsporidian genus Brachiola, with comparisons of human and insect isolates of Brachiola algerae. The Journal of eukaryotic microbiology 51: 678–685. 15666726 10.1111/j.1550-7408.2004.tb00608.x PMC3109626

[44] Kasickova D, Sak B, Kvac M, Ditrich O (2009) Sources of potentially infectious human microsporidia: molecular characterisation of microsporidia isolates from exotic birds in the Czech Republic, prevalence study and importance of birds in epidemiology of the human microsporidial infections. Vet Parasitol 165: 125–130. 19679398 10.1016/j.vetpar.2009.06.033

[45] Santin M, Cortes Vecino JA, Fayer R (2008) Enterocytozoon bieneusi genotypes in dogs in Bogota, Colombia. Am J Trop Med Hyg 79: 215–217. 18689627

[46] Zhang X, Wang Z, Su Y, Liang X, Sun X, et al (2011) Identification and genotyping of Enterocytozoon bieneusi in China. J Clin Microbiol 49: 2006–2008. 21389159 10.1128/JCM.00372-11 PMC3122652

