# Correction: Microsporidia Detection and Genotyping Study of Human Pathogenic *E. bieneusi* in Animals from Spain

**DOI:** 10.1371/journal.pone.0106017

**Published:** 2014-08-12

**Authors:** The PLOS ONE Staff

Dr. Javier Millán is not included in the author byline. He should be listed as the seventh author, and his affiliation is 7: Faculty of Ecology and Natural Resources, Universidad Andres Bellow, Santiago, Chile. The contributions of this author are as follows: Contributed reagents/materials/analysis tools.
