# Correction: Correction: Microsporidia Detection and Genotyping Study of Human Pathogenic *E. bieneusi* in Animals from Spain

**DOI:** 10.1371/journal.pone.0108086

**Published:** 2014-09-08

**Authors:** The PLOS ONE Staff

There is an error in affiliation 7 for author Javier Millán. Affiliation 7 should be: Faculty of Ecology and Natural Resources, Universidad Andres Bello, Santiago, Chile. The publisher apologizes for the error.

## References

[1] Galván-Díaz AL, Magnet A, Fenoy S, Henriques-Gil N, Haro M, et al (2014) Microsporidia Detection and Genotyping Study of Human Pathogenic *E. bieneusi* in Animals from Spain. PLoS ONE 9(3): e92289 doi:10.1371/journal.pone.0092289 24651457 10.1371/journal.pone.0092289 PMC3961313

[2] The *PLOS ONE* Staff (2014) Correction: Microsporidia Detection and Genotyping Study of Human Pathogenic *E. bieneusi* in Animals from Spain. PLoS ONE 9(8): e106017 doi:10.1371/journal.pone.0106017 10.1371/journal.pone.0092289 PMC3961313 24651457

